# Updating the role of matrix metalloproteinases in mineralized tissue and related diseases

**DOI:** 10.1590/1678-7757-2018-0596

**Published:** 2019-08-30

**Authors:** Cintia Kazuko Tokuhara, Mariana Rodrigues Santesso, Gabriela Silva Neubern de Oliveira, Talita Mendes da Silva Ventura, Julio Toshimi Doyama, Willian Fernando Zambuzzi, Rodrigo Cardoso de Oliveira

**Affiliations:** 1 Universidade de São Paulo Universidade de São Paulo Faculdade de Odontologia de Bauru Departamento de Ciências Biológicas Bauru São Paulo Brasil Universidade de São Paulo, Faculdade de Odontologia de Bauru, Departamento de Ciências Biológicas, Laboratório de Bioquímica, Bauru, São Paulo, Brasil; 2 Universidade Estadual Paulista Universidade Estadual Paulista Júlio de Mesquita Filho São Paulo Brasil Universidade Estadual Paulista Júlio de Mesquita Filho, Campus Botucatu, Rubião Jr, São Paulo, Brasil

**Keywords:** Extracellular matrix, Matrix metalloproteinases, Bone, Vascular calcification, Extracellular vesicles

## Abstract

Bone development and healing processes involve a complex cascade of biological events requiring well-orchestrated synergism with bone cells, growth factors, and other trophic signaling molecules and cellular structures. Beyond health processes, MMPs play several key roles in the installation of heart and blood vessel related diseases and cancer, ranging from accelerating metastatic cells to ectopic vascular mineralization by smooth muscle cells in complementary manner. The tissue inhibitors of MMPs (TIMPs) have an important role in controlling proteolysis. Paired with the post-transcriptional efficiency of specific miRNAs, they modulate MMP performance. If druggable, these molecules are suggested to be a platform for development of “smart” medications and further clinical trials. Thus, considering the pleiotropic effect of MMPs on mammals, the purpose of this review is to update the role of those multifaceted proteases in mineralized tissues in health, such as bone, and pathophysiological disorders, such as ectopic vascular calcification and cancer.

## Introduction

Bone is a specialized, vascular, and dynamic connective tissue in constant remodeling to maintain physiological ion homeostasis, give support and protection for soft tissue, and be a reservoir of ions important to vertebrates.^1^^–^^3^ Mechanistically, bone remodeling requires a coordinate and dynamic relationship between deposition/degradation of extracellular matrix (ECM), through growth factors and other signaling molecules, which results in ECM remodeling—an important prerequisite for cell adhesion, migration, proliferation, differentiation.^4^ It is well known that osteoclasts resorb the mineralized matrix and further promote the remodeling of the organic fraction of the bone, while, conversely, osteoblasts are responsible for bone formation by depositing specialized ECM components prior to mineralizing it properly^2^. The balance between those specialized cells is crucial for maintaining appropriate bone mass, and the lack of this synchronism contributes to the occurrence of bone diseases, such as osteoporosis and Paget's. To date, ultimate bone cellular differentiation is known to depend on well-orchestrated communication between formation and resorption events.^2^^,^^5^^–^^7^

**Figure 1:**
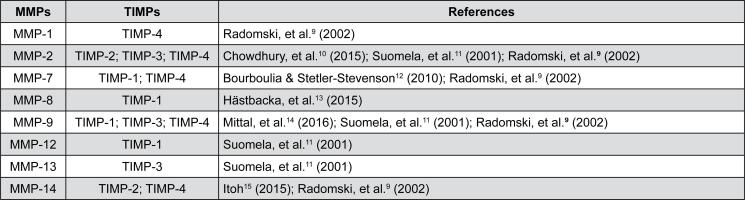
List of major MMPs involved in bone repair processes and their tissue inhibitors (TIMP)^9^^–^^15^

More specifically, during bone repair after trauma, bone healing depends on interactions between specific signaling inflammatory cytokines and non-resident and eventual cells (such as polymorphonuclear leukocytes and monocyte-macrophage lineage),^8^ requiring ECM remodeling by specific matrix metalloproteinases (MMPs). Generally, MMPs are an important family of zinc-dependent endopeptidases and are the major class of enzymes responsible for the degradation or resorption of all ECM components (Figure 1).^9^^–^^15^ MMP targets include other proteases, protease inhibitors, blood coagulation factors, chemotactic molecules, latent growth factors, binding protein growth factor, cell surface receptors, and cell adhesion molecules.^16^^–^^19^ At this stage, bone remodeling is necessary for the homeostasis of systemic calcium release, bone turnover, and repair/regeneration of injured bone.^20^

**Figure 2:**
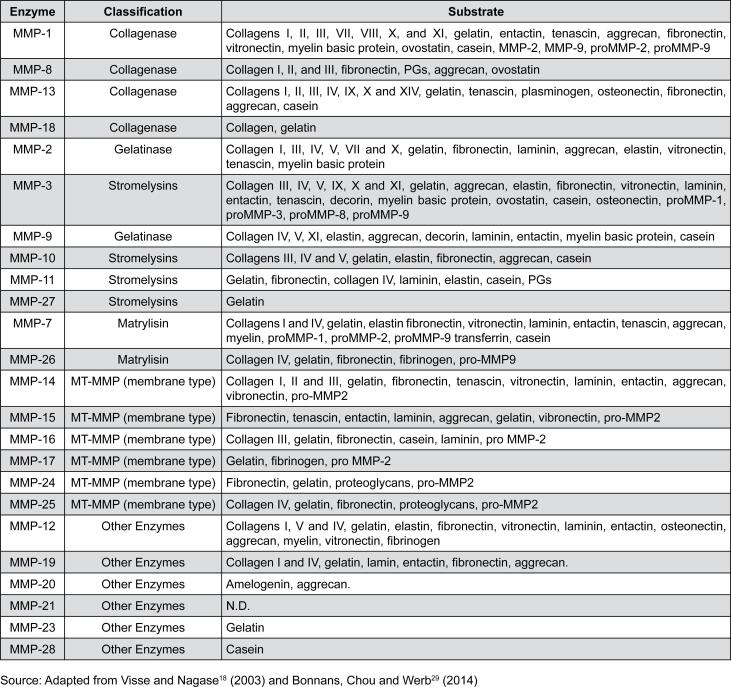
List of MMPs, according their classification and substrate^18^^,^^29^

In turn, MMPs are important regulators of the cellular and physiological processes affecting crucial biological processes, such as angiogenesis, morphogenesis, tissue repair, and are decisive tools for the occurrence of some diseases, such as cancer, cardiovascular disorders, arthritis, among others.^21^^–^^23^ Particularly, MMP-2 plays a role in bone embryonic development, tissue repair, and tumorigenesis,^24^ while MMP-7 stimulates bone differentiation and extracellular matrix degradation,^25^ and MMP-9 seems to be involved in osteoclast-based bone remodeling.^25^^,^^26^ Generally, MMPs are secreted in latent form, such as pro-enzymes or zymogens, and therefore require proteolytic activation occurring after the precise removal of the inhibitory pro-peptide. This biochemical processing occurs by breaking the link between the cysteine-containing thiol group and the zinc in the catalytic domain^27^^,^^28^, resulting in an optimal conformational structure for exerting catalysis mechanisms. MMPs are classified respecting their structure and/or their specific substrates, resulting in a family of 25 members actually (Figure 2).^18^^,^^29^ Conversely, TIMPs are responsible for controlling the breakdown of ECM components by negatively modulating MMPs and they are involved in a range of important biological phenomena and pathological events such as inflammation and tumor invasion. It is known that the TIMPs range of activities can be even wider if we consider their effect on the inhibition of several ADMs and ADMTSs (disintegrins and metalloproteinases).^19^^,^^30^ Additionally, the human genome encodes four genes for TIMPs (TIMP-1 to 4) and have approximately 40% of conserved sequences among them. More specifically, TIMP-2 and TIMP-4 have the most similar sequences with 50% similarity.^19^^,^^30^ The structure of the N-terminal region is highly conserved in all TIMPs that can bend as a separate unit performing its function in inhibiting MMPs.^18^^,^^31^

Because of the classic role of TIMPs, which control extracellular matrix proteolysis (ECM) through the endogenous inhibition of MMPs, it is suggested that increased TIMP results in either accumulation of ECM or fibrosis, while the decrease in TIMP leads to an intense matrix proteolysis.^32^ Thus, ideal tissue remodeling requires a balance between MMPs and TIMPs. The inhibitory activity of TIMPs may be important in inhibiting malignant tumor progression leading to invasion and metastasis.^33^ Regardless of inhibition of MMP, TIMPs act as signaling molecules with cytokine-like activities, thus influencing various biological processes, including cell growth, apoptosis, differentiation, angiogenesis, and oncogenesis.^34^

**Figure 3:**
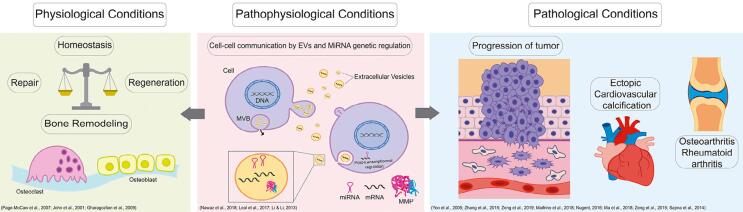
Matrix metalloproteinases are involved in several biological mechanisms, mainly in bone repair and regeneration, which occurs during the homeostasis of MMPs when the osteoclast performs their role in reabsorption and the osteoblast forms a new matrix. In contrast, the MMPs act in pathological conditions such as progression of tumor, bone diseases as OA or RA and ectopic cardiovascular calcification. Pathophysiological conditions have been observed showing that the action of EVs and miRNAs had implicated in the regulation of MMPs acting in others functions in the organism^21^^–^^23^^,^^36^^–^^46^

Bone remodeling and repair comprehend an extraordinarily complex sequential mechanism requiring dynamic and intense ECM components breakdown and drive cell fates involved with adhesion, controlling this mechanism and their regulation is proliferation, differentiation mechanisms, such as well decisive to adequate bone healing. Because MMPs as releasing signaling active molecules during the participate in almost all phases of bone repair, they remodeling process of the hematoma tissue. In this are important biomarkers of this very complex scenario, MMPs are particularly important members and coordinated process.^16^^,^^35^ To the best of our knowledge, this review updates the role of these endopeptidases in mineralized tissue and their effect on pathophysiological conditions (Figure 3).^21^^–^^23^^,^^36^^–^^46^

## MMPs are important mediators of bone physiology

Bone tissue is a very dynamic tissue and requires a repertoire of enzymes capable of degrading the organic fraction of the bone matrix, thus, the action of MMPs and their inhibitors have physiological relevance. The absence of some molecules, such as MMP-9, MT1-MMP (MMP-14), or MMP-13 during the skeletal development results in severe abnormalities in the bone growth plate of long bones, impairing normal bone formation. The ECM remodeling requires specific activities of MMPs. The literature reports an increase in MMPs 2, −3, −9, −13, in osteoblast that leads to bone resorption by stimulating interleukin-1 and - 6.^4^^,^^24^

**Figure 4:**
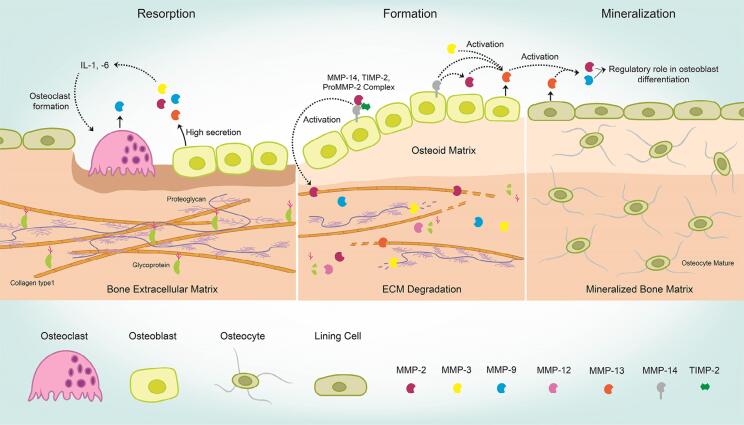
Osteoblast cells release MMPs (as −2, −3, −9 and −13), which have an important role in bone metabolism, activating osteoclast cells and consequently initiating matrix degradation. Afterwards, several matrix metalloproteinases work in coordination in order to form new ECM. Thus, a new mineralized matrix is formed through regulatory mechanism of cells and MMPs^4^^,^^20^^,^^24^^,^^47^

The role of each MMP involved in bone remodeling is not fully clear, but it is known that MMP-2, −13, and −14 plays an essential role. In fact, due to the proteolytic activity of the bone matrix, regulation bioavailability of soluble RANKL, cell–cell interaction, coupled with bone resorption to bone formation, intercellular communication in bone cells, and cell– ECM interaction occurs.^20^ According to Mizutani, et al.^4^ (2001), MT1-MMP actives MMP-2, cleaving the pro-peptide latent form, and MMPs 2; −3; −9; −13, and MT-MMPs play an important role in both formation and bone resorption (Figure 4).^4^^,^^20^^,^^24^^,^^47^ In addition, our group has shown a molecular mechanism involved in osteoblast differentiation *in vitro*, requiring the expression of MMP-9, with higher expression on the 28^th^ day.^3^ Furthermore, this study showed that both MMP −2 and −9 were regulated during osteoblast differentiation and apparently TIMP-2 was essential in the late osteoblast maturation. As seen, recent studies highlight the important involvement of proteases in the extracellular matrix solubilization process, their role in determining where and when the reabsorption will start, and its relationship with new bone formation. However, little is known about the molecular details involved in such process, which makes research in this field focused on the discovery and identification of proteinases.^47^

In addition to TIMPs, another endogenous MMP inhibitor was discovered recently: RECK (“reversioninducing cysteine-rich protein with Kazal motifs”).^48^ RECK is a membrane-anchored glycoprotein capable of negatively regulating peri-cellularly MMPs and compromises ECM remodeling, triggering signaling pathways, such as Notch, which is essential for the process of angiogenesis.^3^^,^^49^ Because of this, RECK has been considered a prognostic marker and potential therapeutic macromolecule in solid cancers. However, although some progress has been achieved highlighting the role of RECK in cancer, our knowledge on their role in bone is scarce. Updating the literature in this regard, Accorsi-Mendonca, et al.^47^ (2008) evaluated the expression of matrix metalloproteinase-2, −9 and RECK through immunohistochemistry during the well-characterized alveolar bone regeneration model: their results show that active and/or inactive forms of MMP-2 and MMP-9, and RECK are differentially expressed by osteogenic cells and connective tissue such as bone formation, maturation, and remodeling. Also concerning craniofacial structures, Demarchi, et al.^50^ (2010) showed differential, temporal, and spatial expression of RECK during the development of secondary palate. This distribution was compared with the expression of MMP-2, MMP-3, and MMP-9, and they suggested RECK might reorganize the epithelium and mesenchyme palatine blades. Additionally, we have also dedicated to evaluate the involvement of MMPs and their inhibitors in adaptive osteoblast processes to biomaterials, and it is clear that ECM remodeling is a prerequisite to drive cell adhesion on biomaterials surfaces.^51^^–^^55^

Although bone healing is a physiological mechanism able to regenerate enough tissue to heal small injuries, larger lesions require therapeutic alternatives to support bone growth. In this context, bone tissue engineering emerges as an interesting field within regenerative medicine, providing strategies to regenerate lost bone and thereafter restoring their physiological function.^3^ In this aspect, the cooperation of biology, chemistry, physic, engineering, and biomedicine is established to evaluate biomaterials serving as scaffold for bone growth. The studies have focused on classifying metallic, organic, and inorganic materials regarding international rules of biocompatibility. Thus, we have tested the hypothesis that MMP-9 may be involved in tissue remodeling in response to xenogeneic hydroxyapatite, due to MMP-9 role in the breakdown of extracellular matrix components. The presence of CD68+ cells in response to natural hydroxyapatite was also investigated. The results suggest that tissue remodeling in response to natural hydroxyapatites requires MMP-9 expression and CD68+ cell recruitment.^3^ This was also extended to the MMP2 role in reactive tissue in response to granules of hydroxyapatite.^56^ To date, CD68 is a macromolecule widely found in macrophages and here they were discussed to drive osteoclastogenesis.^3^

Thus, it is essential the balance between metalloproteinases and their inhibitors to understand the mechanisms involved in bone ECM remodeling and keep bone quality. If certain MMPs are not expressed during bone formation, the process does not take place accordingly, e.g.: inflammatory conditions, human genetic mutations, metabolic disorders, bone tumors, and metastasis.^20^

## A sophisticate manner to release MMPs: extracellular vesicles

Over the last years, it has been proposed that MMPs are released by using the sophisticate extracellular vesicles (EVs) strategy. EVs are nano- and micro-sized membrane-bound vesicles such as exosomes and microvesicles, secreted by many types of cells that can transport cargo, including proteins, lipids, nucleic acids, and membrane receptors from donor cells.^36^^,^^57^^,^^58^ It has also been observed that EVs share structures homologous to those with matrix vesicles (MVs), which are characteristic of matrix mineralization, such as mineralization of cartilage, bone and dentin, for example.^36^ EVs are released by plasma membranes, differentiating chondrocytes growth, odontoblasts, and osteoblasts.^36^^,^^59^ In general, these MVs contain matrix processing enzymes, such as MMP-2, MMP-9 and MMP-13, which play an important role in matrix remodeling, mainly degradation of proteoglycans, and allow calcification.^60^^,^^61^ Additionally, the activation of TGF-β takes place through MVs containing MMP-3, synthetized by growth plate-resident chondrocytes and the production of matrix vesicles is induced by phosphate through extracellular signal-regulated kinases ERK1/2 pathway.^36^ Until now, MVs and EVs exhibit similar activities for mineralization of ECM, but are biological distinct entities.^62^

The secretion and high number of EVs into biological fluids represent an exceptionally large interactive surface area that establishes contact with cells and molecules in the extracellular microenvironment.^63^ Therefore, the presence of matrix processing enzymes in EVs and their secretion along the entire cell periphery provides an additional layer of interactome and play an active role in pathological and physiological processes.^36^ According to Liu, et al.^64^ (2018), bone-remodeling micro-environment, bone-derived EVs contains specific osteogenic proteins, such as bone morphogenetic protein 1-7, alkaline phosphatase (ALP), eukaryotic initiation factor 2, and noncollagenous matrix proteins, such as bone sialoprotein, osteopontin, osteocalcin, and osteonectin. Moreover, EVs contain matrix-degrading enzymes such as MMPs, heparanases, hyaluronidases, extracellular matrix metalloproteinase inducer (EMMPRIN), aggrecanases, such as adamalysin metalloproteinases having disintegrin and thrombospondin domains (ADAMTSs), and TIMPs, among others.^36^ Altogether, these findings led us to reinforce the role of these microvesicles in delivering MMPs at the outside compartment. This process seems to be essential in the signaling of molecules proteolytically involved with mineralization mechanism. EVs are a biological strategy to deliver ECM-related proteases.

In fact, for their conversion into functionally active MMPs, membrane-type MMPs are internalized to the endosomes and are recycled to the plasma membrane.^65^ However, instead of recycling, the membrane-like proteins of the endosomes might be cleaved, converted into functionally active soluble forms, and packaged in the intraluminal vesicles and secreted via EVs,^66^ e.g.: after MMP14 internalization in the endosomes, this molecule was not recycled back into the plasma membrane, but rather packed into EVs and secreted into the extracellular environment to act as a functionally active soluble.^67^ Lastly, the detection of these secreted molecules in circulating EVs of various body fluids makes them an ideal source of disease biomarkers.^68^^,^^69^

The understanding of how extracellular vesicles act in intercellular communication as paracrine mediators in normal conditions as well as cause the progression of diseases has aroused interest of researchers. Cells are known to use cytoplasmic extensions that act as open-ended channels called tunneling nanotubes (TNTs) to connect cells at a long distance and make the exchange of cytoplasmic material easy. Thus, in the future, EV and TNTs should be used as drug-delivered vector against many diseases.^70^

## The other side of the coin: role of MMPs in tissue disorders

### Cancer

Signals coming from the outside at the surrounding micro-environment, such as growth factors, extracellular matrix (ECM), adjacent stromal cells, cytokines, and chemokines, profoundly influence stem cell fates.^71^ The complete understanding of the extracellular matrix remodeling is crucial to deliver signals guiding cellular phenotypes and, as expected, MMPs are discussed as important proteases in this context. In conjunction, MMPs play several key roles in the metastasis mechanism and might contribute to all stages of tumor progression and implantation.^71^^,^^72^

Molecularly, there are more than 500 genes encoding proteases or protein-like proteases in the human genome.^73^ However, among all proteolytic enzymes potentially associated with tumor invasion, members of the MMP family have emerged as important proteases mainly because of their ability to degrade almost all components of the ECM and basement membrane, allowing cancer cells to penetrate, infiltrate in the underlying stromal matrix,^74^^,^^75^ and initiate the metastasis mechanism as well. The process of invasion and metastasis of tumor cells occurs basically through mechanisms of cellular mobility accompanied by degradation of the basement membrane and components of the extracellular matrix.^71^ MMPs, especially MMP-2 and MMP-9 (both known as gelatinases A and B, respectively), are involved in several mechanisms contributing to cell adhesion and invasion, and further tumor progression.^39^^,^^40^^,^^76^ The action of proteases assures the breakdown of barriers of ECM matrix components, mainly collagen, laminins, and proteoglycans, and favors cell invasion, thus driving the pathway to orchestrate invasive cancer cells.^71^ In conjunction, MMPs are able to guide adherent cell phenotype by interfering on cell adhesion, also resulting in an adaptive process of cancer cells by interacting either with each other or with the components of the substrate, reinforcing the cell-cell and matrix-cell interfaces, respectively.^72^ Lastly, MMPs also modify substrates other than ECM structural molecules. MMPs activate proteinase activated receptors (PARs) by the cleavage of their extracellular domains, promoting tumor progression and favoring the invasive phenotype. Altogether, growth factor-related receptors, cell adhesion molecules, chemokines, cytokines, apoptotic ligands, and angiogenic factors are just few examples of the diversity of substrates recognized by MMPs.^74^^,^^75^

**Figure 5:**
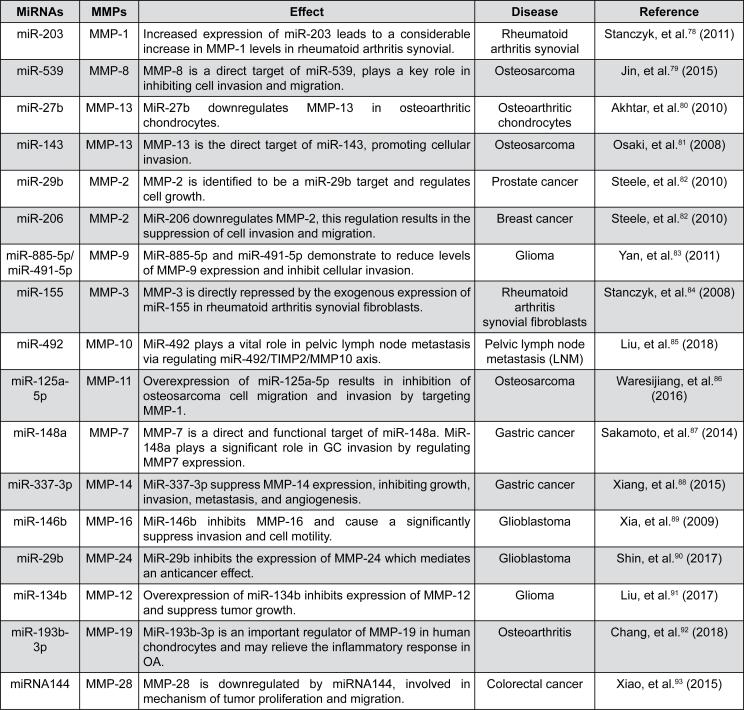
Summary of the miRNAs effect over MMPs in some diseases^78^^–^^93^

In health, MMP-2 (gelatinase A) is involved in many physiological factors and processes, including healing and repair as discussed earlier here. However, in tumor tissues such as osteosarcoma, the MMP2 gene is overexpressed and contributes to the acceleration of extracellular components turnover and breakdown. Overall, MMP expression plays a crucial role in the development of osteosarcoma and consequently developing pulmonary metastasis in many individuals.^39^^,^^40^ In this way, Zhang & Zhang^40^ (2015) found MMP-2 involvement was higher in the individuals presenting osteosarcoma than in the control group. Moreover, the overall survival rate was higher in individuals with negative MMP-2 expression, and the group presenting negative expression of MMP-2 lived longer than the group with those positive expressions. In addition, they show that abnormal expression of MMP-2 was associated with pulmonary metastasis. Fang, et al.^77^ (2000) found many other roles of MMP-2 in tumor progression, such as their involvement with EGFR signaling and integrin, which leads to cell migration and the shift to an angiogenic phenotype. Thus, it is clear that MMP develops a pleiotropic effect on tumor installation by remodeling ECM and activating signaling molecules, impacting cell adhesion and metastatic phenotype (Figure 5).^78^^–^^93^

Studies suggested that MMPs are involved in initial step of ECM degradation during metastatic process.^5^^,^^22^^,^^40^^,^^72^ MMPs have a complex role and mediated degradation of ECM components might generate inhibitors of angiogenesis, promoting stimulate cell migration and/or invasion, and release activated cytokines stored in ECM. Type I collagen comprises 90% of the organic matrix, in bone matrix (BM) and in cancer dissemination, the BM must be broken down mainly in interstitial collagenase, MMP 1.^94^

Okuyama, et al.^94^ (2008) suggest that MMP-1 play an important role in maintaining high bone metastatic potential in their study. Moreover, researchers reported that osteoclasts stimulated by cancer cells, directly or not, play an important role in bone resorption and in breast cancer condition, PTH-rP (parathyroid hormone-related protein) released by cancer cells stimulate the formation and resorptive activity of osteoclasts. Prostate cancer shows a tendency to involve bone and a marked response of the bone to the presence of bone cells, and there is a general consensus that bone formation and bone breakdown are present within metastatic deposits.^95^

Nemeth, et al.^95^ (2002) report that bone is the most frequent organ affected and the presence of metastatic cells leads to an increase in bone matrix turnover. They propose that stimulation of bone matrix turnover by metastatic cells may be responsible for a prostate cancer tendency towards growing up within the bone environment. The MMPs appear to play critical roles in prostate cancer metastasis and bone matrix turnover, it is hypothesized that inhibition of MMP activity may disrupt the cycle of bone matrix turnover and tumor cell growth^94^^,^^95^. The communication between tumor cells and bone cells increase bone metabolism, and the release of stimulatory substances during bone matrix turnover enhances growth of cancer cells that have colonized bone.^95^

## MiRNAs (miRs)-based post-transcriptional processing of MMP and cancer development

The miRs encompasses approximately 17–25 nucleotides in length and are small endogenous non protein coding RNAs whose function is involved in the regulation of post-transcriptional gene via translational repression or the degradation of their mRNA targets.^37^^,^^38^ In summary, most mammalian miRNAs use a second mechanism of gene regulation that does not involve the cleavage of their mRNA targets. miRs exert their regulatory effects by binding to imperfect complementary sites within the 3′ untranslated regions (UTRs) of their mRNA targets and they suppress target-gene expression post-transcriptionally at the level of translation by means of a RISC complex that is similar, or possibly identical to the one that is used for the RNAi pathway.^96^ The activities of miRs are essential for gene regulation during skeletal development and homeostasis, and their adequate expression is essential for bone formation and maintenance. Furthermore, miRs regulates signaling pathways such as TGFβ, BMP, and Wnt that are involved with dynamics and bone disorders, emphasizing their potential therapeutic role.^97^ Thus, miRs involved in bone pathologies such as osteosarcoma and cancer metastasis pose as interesting targets for possible intervention.^98^ However, the *in vivo* functions of individual miRNAs in maintaining bone homeostasis and resulting in disease, clinical applications, and expression need to be better addressed.^99^

Since MMPs develop crucial roles in several physiological processes in health and disorders, it is urgent to comprehend their molecular mechanism, such as those promoted by miRs-based mechanism to molecular processing MMPs. The miRs are involved in multiple functions, such as proliferation, apoptosis, senescence and differentiation, playing an indispensable role in tumor initiation/progression, metastasis, invasion in many types of cancer, and the post-transcriptional regulation of MMPs is modulated by miRs^100^^–^^103^ (Figure 5). Additionally, polymorphisms are also important in genes encoding MMPs and they lead to changes in MMP expression patterns in cancer.^104^ The relations between the MMP overproduction in tumor or stromal cells and the progression of cancer led to the development of clinical trials testing a series of inhibitors intended to block the proteolytic activity of these enzymes. However, the application of MMP inhibitors in patients was unsuccessful because some MMPs may play a paradoxical protective role in tumor progression along with the identification of new roles of MMPs in the initial stages of cancer.^74^^,^^75^

## MMPs and specific inflammatory landscape

MMPs have been listed in many disorders. In rheumatoid arthritis (RA) and osteoarthritis (OA), the MMP's activities are directly related to cartilage degradation. According to Xue, et al.^105^ (2014), endogenous MMP-2 or MMP-9 collaborate to survival, proliferation, migration, and invasion of RA synovial fibroblast. In addition, MMP-9 stimulates RA synovial fibroblast-mediated inflammation and degradation of cartilage, contributing to joint destruction. The overproduction of MMP-13 by chondrocytes during onset and in progression of OA, promoting the extracellular matrix degradation.^43^^,^^44^

In the study by Zeng, et al.^45^ (2015), protein levels of MMP-1, MMP-2 and MMP-9 were higher in patients with OA compared to the control group, and the ethnicity showed that levels of MMP-1 and MMP-2 were higher in Asians with OA compared to the control group. However, the levels of MMP-9 in OA patients were higher than that of control group, Asians and Caucasians.^45^ Additionally, the matrix metalloproteinases are responsible for collagen and ECM degradation, in periodontal disease.^106^ MMP-8 and MMP-13 related to tissue destruction followed with significant contribution of MMP-9 and MMP-14.^46^

## MMPs and ectopic vascular calcification

As above-mentioned, mineralization is not an exclusive mechanism of bone. more specifically, ectopic vascular calcification has become an important topic in public health and over the last years, several studies have shown the important involvement of MMPs and TIMPs in cardiovascular diseases, including coronary artery disease,^107^ myocardial infarction,^108^ atherosclerosis,^109^ and ischemic stroke.^110^^,^^111^ In cardiovascular diseases, MMPs act in the weakening of vessels since they are able to degrade all their major components, essential for the maintenance of vascular tonus.^112^ The main MMPs involved in cardiovascular diseases are MMP-2 and MMP-9.^41^^,^^42^

We have shown recently that the mechanostimulation of smooth muscle cells provokes hypermethylation of the TIMP1 promotor, resulting in a decrease of TIMP1 transcription and translation in conjunction with an increased matrix remodeling enzymatic activity. Therefore, we have defined a novel-signaling pathway responsible for the physiological adaptation of vessel smooth muscle to changes in mechanical pressure, which are potentially relevant for defining novel avenues for the rational treatment of diseases.^113^ Chemical-motivated inhibitors of MMPs have been shown to be promising strategies for cardio-protection. Bencsik, et al.^114^ (2014) found that the inhibitor of MMP ilomastat was able to moderately inhibit the expression of MMP-2 in rats with induced myocardial infarction, which led to the conclusion that its function is sufficient to confer cardiovascular protection. A physiological imbalance between MMPs and TIMPs may also result in disorders involving the heart and blood vessels.^91^^,^^109^ On the other hand, MMP inducers appear to contribute with the progression of cardiovascular diseases. EMMPRIN, one of these inducers, has been implicated in the development of vascular diseases, including the pathogenesis of myocardial infarction.^108^ Understanding the role of MMPs and their endogenous or synthetic inhibitors in the cardiovascular system may contribute to the modulation of these factors in the case of heart disease^115^ and vascular disorders such as vascular calcification, which is an important etiology for disturbance of the blood flow reaching high blood flow pressure locally and systemically. Of course, the calcification process requires a dynamic remodeling of ECM prior to the deposition of inorganic components, and there has been discussion on whether this process repeats osteogenic features or not ^4^^,^^20^^,^^35^^,^^47^.

## MMP inhibitors as an alternative for anti-cancer therapy

Considering the importance of MMPs roles in modulating ECM rearrangement and consequently affecting cell viability, the knowledge about their molecular processing is urgent. Many MMP inhibitors have been designed and synthesized seeking to treat malignant tumors or other disorders, which requires intense ECM remodeling in order to provide changeable scaffold during the adhesion and migration stages. Among these drugs, peptidomimetics, non-peptide, tetracycline derivatives and bisphosphonates can interfere in MMP activities and they are expected to modulate cancer progression. Thus, the function of MMP inhibitors has been sought as an alternative form of anticancer therapy.^116^

The literature is controversial regarding the use of several synthetic types of MMP inhibitors. The study by Santos, et al.^117^ (1997) evaluated the action of some MMP inhibitor-related compounds (AG3287, AG3293, AG3294, AG3296, AG3319 and AG3340), using lung carcinoma as experimental model, and found that AG3340 was the most effective in inhibiting neoplastic growth. However, other compounds, such as AG3293 and AG3294, did not present anti-tumor action. The selectivity of those inhibitors must also be considered: high selectivity of individual MMPs has major biomedical importance. It is known that both MMP-2 and MMP-9 are important therapeutic targets.^118^^,^^119^ Jha, et al.^118^/EndNote>(2016) evaluated the mechanism of inhibition of epigallocatechin-3-gallate (EGCG)-mediated MMP-2. Additionally, they concluded that the EGCG targets fibronectin II repeating regions 1 and 3 of MMP-2, binds the amino acids that constitute the exosite of this enzyme and hinders the proper positioning of the substrate. EGCG is an important polyphenol of green tea with a potential chemotherapeutic agent demonstrating anti-metastatic and MMP inhibitory activities in addition to various other already known biological activities.^118^^,^^120^ Additionally, the effects of EGCG were also evaluated in anti-proliferation and anti-migration against bladder cancer SW780 cells for both *in vitro* and *in vivo*. Treatment of EGCG resulted in significant inhibition of cell proliferation by induction of apoptosis, with no toxicity to cells of the normal bladder epithelium. When tested *in vivo*, EGCG significantly decreased tumor volume in mice with SW780 tumors (tumor weight decreased 68.4%). In addition, it regulated the expression of nuclear factor kappaB (NF-kB) and MMP9 at both protein and mRNA levels in tumor cells and SW780. EGCG was shown to be effective in inhibiting the proliferation and migration of SW780 cells and inhibited SW780 tumor growth by down-regulation of NF-κB and MMP-9.^120^

Inhibition of MMP does not only intend to be used in cancer therapy but also in other diseases. In a recent study, a new mercaptosulfonamide-based MMP inhibitor, YHJ-7-52, was evaluated in the differentiation of human mesenchymal stem cells (hMSCs) into adipocytes and in the accumulation of lipids. The YHJ-7-52 was shown to be an effective regulator of hMSC adipogenesis. Inhibition of MMP was also able to suppress lipid accumulation in adipocytes co-treated with Troglitazone. These authors suggest that MMP inhibitors might be used as molecular tools for research on adipogenesis and treatment of obesity. Therefore, inhibition of MMP not only provides cancer therapy, but also a great potential for adipogenesis and treatment of obesity.^121^

Wylie, et al.^122^ (1999) found that Batimastat (BB-94) is a MMP inhibitor and Maekawa, et al.^123^ (2000) evaluated the action of MMI-166, an inhibitor with selective spectrum for gelatinases, on several models of metastasis. They found that this drug significantly reduced growth of tumor metastasis in the lung, liver, and peritoneal cavity *in vivo*, although it does not affect neoplastic cell growth *in vitro*, reinforcing the limitation of these *in vitro* models to some anti-tumor studies. Thebiology of MMPs is thus very complex and has been listed within several pathologies, and as such further studies are necessary evaluating these proteases as promising therapeutic strategies.^116^ However, it is important to note that further investigation is urgent for determining the clinical efficacy and safety of these many suggested synthetic MMP inhibitors in humans.

Thus, to the best of our knowledge, the understanding of MMPs biology at a physiological, cellular, and molecular level should drive pharmacological studies focusing on the chemical inhibition of pathological processes. Therefore, new strategies to better elucidate these mechanisms are necessary, by complementing advancements in the structural and biochemical understanding of MMPs biology within mineralized tissues and this update on understanding their role in the body claims for preclinical analysis and further clinical trials.

## Conclusion

MMPs are widely distributed in physiological processes and in conjunction they drive important biological mechanisms such as angiogenesis, embryogenesis and dynamic tissue remodeling throughout growth and aging. However, the main role of MMPs is shared with important etiology of diseases, such as bone and vascular disorders and cancer. Thus, the understanding of the biology of MMPs should be considered chemical routes to synthetize drugs capable to efficiently and selectively affect MMP activities. This review updated the knowledge on the importance of MMPs and their inhibitors in distinguishing health and diseases conditions, reporting the modulation of their molecular processing and activities and reinforcing the need to use their biochemical mechanism lessons as templates for the chemical development of new drugs aiming for mostly efficient therapies.
